# Factors associated with uncontrolled asthma in adult asthmatics in Kinshasa, Democratic Republic of Congo

**DOI:** 10.1371/journal.pone.0215530

**Published:** 2019-04-18

**Authors:** Benoit Obel Kabengele, Jean-Marie Ntumba Kayembe, Patrick Kalambayi Kayembe, Zacharie Munogolo Kashongue, Didine Kinkodi Kaba, Pierre Zalagile Akilimali

**Affiliations:** 1 Unit of Pneumology, Department of Internal Medicine, University Clinics of Kinshasa, Medical School, University of Kinshasa, Kinshasa, Democratic Republic of Congo; 2 Department of Epidemiology and Biostatistics, School of Public Health, University of Kinshasa, Kinshasa, Democratic Republic of Congo; Forschungszentrum Borstel Leibniz-Zentrum fur Medizin und Biowissenschaften, GERMANY

## Abstract

**Background:**

The ultimate goal of asthma treatment is long-term control. Uncontrolled asthma is a major public health problem worldwide, but there is a lack of data on asthma control and its causes in the Democratic Republic of Congo (DRC).

**Objective:**

To determine the socio-demographic, environmental, clinical, and biological factors and comorbidities associated with uncontrolled adult asthma in Kinshasa, DRC.

**Methods:**

We performed a cross-sectional study of 216 male and female asthmatics aged 18 and over consecutively recruited from tertiary clinics and the community in Kinshasa between June 2017 and February 2018. For each subject, socio-demographic, clinical, para clinical and environmental data were recorded. Forced Expiratory Volume in one second (FEV_1_) values were obtained by Spirometry, allergen testing performed using the skin prick test, serum vitamin D levels measured by radioimmunoassay, and asthma control assessed using the asthma control test (ACT) score. Multiple logistic regression identified factors associated with uncontrolled asthma.

**Results:**

The average age of participants was 45.2 (SD 17.6) years, 74% were female, and 42% had a low educational level. Among all asthmatics, the prevalence of uncontrolled asthma was 56%, abnormal serum vitamin D level 95%, abnormal FEV_1_ 65%, sensitization to two allergens (cat dander and dust mites) 18%, sleep disorders 37%, and heartburn 60%. Male (aOR 2.24; 95% CIs 1.04–4.79), low educational level (aOR 3.26; 95% CIs 1.54–6.67), sensitization to both cat dander and dust mites (aOR 2.67; 95% CIs 1.16–6.14), FEV_1_ ≤80% (aOR 2.07; 95% CIs 1.08–3.96), abnormal serum vitamin D level (aOR 5.11; 95% CIs 1.17–22.33), sleep disorders (aOR 1.96; 95% CIs 1.04–3.71), and heartburn (aOR 2.02; 95% CIs 1.04–3.92) were significantly associated with uncontrolled asthma.

**Conclusion:**

Uncontrolled asthma is common in Kinshasa, and these factors associated with uncontrolled asthma may be considered as targets for future intervention strategies.

## Introduction

Asthma, a heterogeneous disease with a complex and multifactorial physiopathology, is a major global public health problem. The estimated global prevalence of asthma is 334 million, including 50 million affected individuals in Africa, and 9% of young adults aged 18–45 have experienced asthmatic symptoms [1–3]. In Kinshasa, the capital of the Democratic Republic of Congo (DRC), the reported prevalence of asthma in adults is 6.9%, with even more (7.3%) reporting chest wheeze at some point in their lives [4]. Asthma affects affected individuals’ quality of life and increases hospitalizations and medication use [5].

Clinical evaluation criteria for asthma have evolved in recent years, positioning assessment of asthma control—both symptomatic and for reducing future risk—as the central element in asthma management [1, 6]. Asthma can be deemed under control when day and night time symptoms or exacerbations are absent and patients experience unlimited daily activity and normal respiratory function [1, 7]. Few previous studies have validated asthma control test (ACT) as an assessment tool for asthma control [1, 8, 9].

Nevertheless, uncontrolled asthma remains a major public health problem. In developed countries, asthma is insufficiently controlled in 40–70% of patients [10, 11, 12]. Africa is no exception; in Cameroon, for example, 42% of adult asthmatics are inadequately controlled [13]. Factors known to influence asthma control include socio-demographic characteristics, psychosocial factors, asthma severity, adherence to treatment, drug inhalation technique, and exposure to infectious agents (especially viruses) and allergens (indoor and outdoor pollutants). Comorbidities (high blood pressure, chronic sinusitis, gastroesophageal reflux disease, obesity, hypovitaminosis D) also increase the risk of poor asthma control [8, 14, 15].

However, asthma control data from low-income countries are scarce, and it is difficult to generalize data obtained in other parts of the world to specific ethnic and social contexts. There are no studies on asthma control in the DRC. Here we fill this knowledge gap and report the socio-demographic, environmental, biological and clinical factors, and comorbidities associated with uncontrolled asthma in adult asthmatics in Kinshasa. Our overall goal is to identify context-specific risk factors to guide targeted management strategies that are more adapted for DRC.

## Material and methods

This was a cross-sectional study conducted between June 14, 2017 and February 27, 2018 in Kinshasa. Given the lack of asthma registers in health facilities, including information on patients with asthma, the University Clinics of Kinshasa and some parishes and revival churches in Kinshasa were selected for subject recruitment.

The study population consisted of asthmatics aged >18 years who freely agreed to (i) answer questions, (ii) skin allergy testing, (iii) venepuncture for a blood sample for determination of vitamin D levels, and (iv) spirometry. Asthma was considered to be present in any person reporting to have asthma and who was taking asthma medication or any person recognized as asthmatic by a health professional. Pregnant asthmatics, people on antihistamines, those taking vitamin D, those with an exacerbation of asthma, and those being followed for chronic renal failure were excluded from the study.

Interviews were conducted in Lingala (vernacular language in Kinshasa) or French by 4 physicians of Pneumology Department, who were trained on the ethical issues and the tools.

Several socio-demographic variables were collected from each participant using a questionnaire including age, gender, marital status, education level, household size, and socio-economic level. Asthmatics with less than a high school education were considered to have a "low" level of education, those who had completed high school or vocational training as having a "medium" level of education, and those who had completed higher education or university as having a "high" level of education. The socio-economic level was determined by a wealth index constructed from information on whether or not the study participant owned certain durable goods and on certain housing characteristics. Participants were classified according to the wealth index divided into quintiles from the lowest (first quintile) to the highest (fifth quintile), where the first and second quintiles were classified as "low", the third as "medium", and the fourth and fifth as "high". Environmental data (carpet, dog or cat possession), tobacco exposure, family history of atopy/allergy, and the existence of co-morbidities were also collected.

The body mass index (BMI) was used as a proxy to assess the nutritional status. It was calculated as the respondent’s weight (in kg) divided by his height (in metres) squared. A BMI of <20 kg/m^2^ was considered "lean", 20–24.9 kg/m^2^ "normal", 25–29.9 kg/m^2^ "overweight", and ≥30 kg/m^2^ "obese".

The skin prick test (SPT) was used for allergy testing according to European Academy of Allergy and Clinical Immunology recommendations [16]. Five extracts were tested, namely those from dog and cat dander, house dust mites (*Blomia tropicalis*), molds (*Alternaria alternata*), and egg yolk. Histamine solution (10 mg/ml) and phenol glycerol-saline were used as positive and negative controls, respectively. Allergen drops and controls were placed on the anterior surface of the right forearm at intervals of at least 2 cm, and a sterile disposable needle was used to inject the skin, perpendicular to the skin surface and with identical pressure for each allergen or control. The skin was assessed 10 to 15 minutes after introduction of the allergen into the skin, where a papule ≥3 mm was deemed positive. Allergic sensitization was defined as a positive reaction to at least one allergenic extract.

Respiratory function was assessed by spirometry using a portable MiniSpir spirometer/oximeter device produced by Medical International Research. Several parameters were measured including the forced expiratory volume in the first second (FEV_1_). A respondent with an FEV_1_ >80% was considered to have "normal" lung function and one with an FEV_1_ ≤80% was considered to have "abnormal" lung function [17].

A total 25-hydroxyvitamin D (25-OH vitamin D) radioimmunological assay was performed on a Wallac Wizard 1470 automatic gamma counter calibrated for iodine 125 at the Kinshasa Regional Center for Nuclear Studies on frozen serum at -20°C/-40°C using reagents from Demeditec Diagnostics GmbH (Kiel, Germany). Reagents included standards with concentrations ranging from 0 to 100 ng/ml, control serum, tracer (iodine 125 total vitamin D), tubes coated with 25-OH vitamin D total antibodies, incubation buffer, and wash solution. Results were derived from the standard curve by interpolation. The curve was used to determine the total vitamin D level of all samples measured at the same time as the standards. Serum vitamin D levels ≥30 ng/ml were considered "normal" or "sufficient" and serum levels <30 ng/ml were considered "abnormal": serum levels between 10 and 29 ng/ml were interpreted as "vitamin D insufficiency" and those <10 ng/ml as "vitamin D deficiency" [18].

### Measurement of asthma control

The ACT [1, 8, 9] was used to determine the level of disease control over the previous four weeks by assessing the degree of limitation of physical activity, shortness of breath, asthma symptoms, use of a rescue inhaler, and subjective assessment of asthma control. For each question, five responses ranging from one to five were offered and only one answer was possible for each question. Each question generated a score, and the five scores were summed to obtain a total score. A score ≥20 indicated "controlled" asthma and a score ≤19 indicated "uncontrolled" asthma [1].

### Data analysis

Data were entered into EpiData 3.1. After quality control and consistency checks, data were exported into SPSS 23.0 (IBM Statistics, Chicago, IL) and Stata 13 (StataCorp, College Station, TX) for analysis. Descriptive statistics were used to describe the basic characteristics of the study data. Means and standard deviations (SDs) were calculated for normally distributed continuous variables, while proportions with their 95% confidence intervals (CIs) were calculated for categorical variables. The median was calculated for continuous variables with an asymmetric distribution.

The Z-test was used to compare the proportion of people with uncontrolled asthma according to the presence or absence of co-morbidities. Pearson’s chi-square test or the Fisher’s exact test were used to test for associations between asthma control and each independent variable. We calculated the Pearson correlation coefficient between serum vitamin D level and age. The logistic regression model was used to identify associated factors with uncontrolled asthma and to obtain adjusted odds ratio (aOR) and 95% confidence intervals (CIs). Overall, the following variables were included in the final logistic regression model: age, gender, educational status, socioeconomic status, history of smoking, family history of atopy, sensitization to both cat dander and dust mites, serum vitamin D level, sleep disorders, hypertension, heartburn and FEV_1_. Backward selection was the method used for that final model. We fit the full model on all explanatory variables; only the significant explanatory variables were kept in the final model. Variance-inflation factors (VIF) was estimated to assess multicollinearity. We assessed interaction between socioeconomic status and education status, sensitization and socioeconomic or educational status, as well as serum vitamin D level and age. A significance threshold of α = 0.05 was used for all tests.

### Ethical statement

The Ethics Committee at the Kinshasa School of Public Health, University of Kinshasa approved the study protocol (ESP/CE/030/2017). The study was conducted according to the principles expressed in the Helsinki Declaration. The informed consent form was read aloud to each participant and verbal consent was obtained from each participant. As some of the participants were illiterate, in order to standardize the inform consent process, we decided that the consent was to be verbal but witnessed by a third party, who was there to certify that the consent was read to the participant who freely accepted to participate in the study. A copy of the consent form signed by the witness was given to the participant to keep. The process of obtaining the consent was approved by the ethics committee. No minor was included in this study. Data were collected and analysed anonymously. No personal identifiers of participants were recorded on the survey questionnaire. Respondents were informed that their participation was voluntary. They were free to accept, to refuse to participate or to withdraw at any time without any penalty.

## Results

### General characteristics of the study population

Two-hundred and sixteen adult asthmatics were recruited and voluntarily agreed to participate. Participants were aged 18–88 years with an average age of 45.2 ± 17.6 years. About 15% of participants in the study were under 25 years of age, and 46% were at least 50 years of age. Almost three-quarters (74%) were female, and 45% were in a union. Four out of ten participants had a low level of education (42%) and a low socio-economic level (40%). Over half of participant households (54%) contained at least six people. Fifty-six percent of asthmatics had family history of atopy and 13% were active or smokers who had quit within the last six months (Table 1).

**Table 1:** Sociodemographic characteristics of adult asthmatics in Kinshasa.

Characteristics of participants	n	%
**Age**		
< 25 years	32	14.8
25–49 years	86	39.8
≥ 50 years	98	45.4
**Gender**		
Male	56	25.9
Female	160	74.1
**Marital status**		
Single	83	38.4
Married	96	44.5
Divorced/Separated/Widowed	37	17.1
Educational level*		
Low	90	41.7
Medium	72	33.3
High	54	25.0
**Household size**		
≤ 6 persons	98	45.4
> 6 persons	118	54.6
Socio-economic level**		
Low	86	40.0
Medium	43	20.0
High	86	40.0
**Family atopy**		
Yes	120	55.6
No	96	44.4
**Smoking**		
No, or ex-smoker of > 6 months	187	86.6
Yes, or ex-smoker of < 6 months	29	13.4

*Asthmatics who had not completed high school were considered to have a "low" level of education, those who had completed high school or vocational training as having a "medium" level of education, and those who had completed higher education or university as having a "high" level of education.

**Participants were classified according to the wealth index divided into quintiles from lowest (first quintile) to highest (fifth quintile), where the first and second quintiles indicated a "low" socio-economic level, the third as a "medium" socio-economic level, and the fourth and fifth as "high" socio-economic level.

Almost six out of ten participants (56%) had uncontrolled asthma. Seventy-three percent had allergic rhinitis, 60% heartburn, 37% sleep disorders, 28% hypertension, 27% sinusitis, and 21% allergic dermatosis. Twenty-six percent were overweight, 16% obese, and 14% lean. Spirometry revealed that 65% of asthmatics had an FEV_1_ ≤80%. Only five percent had normal serum vitamin D levels. Regarding sensitization, 38% were sensitive to house dust mites, 24% to cat dander, 18% to dog dander, 8% to molds, and 7% to egg yolk. Additionally, 18% of adult asthmatics were sensitive to both house dust mites and cat dander, two of the most frequently encountered environmental allergens (Table 2).

**Table 2:** Clinical, para clinical, and environmental data for adult asthmatics in Kinshasa.

Characteristics of participants	n	%
Asthma control test (ACT)*		
Controlled asthma	95	44.0
Uncontrolled asthma	121	56.0
**Comorbidities**		
Allergic rhinitis	156	72.6
Heart burn	130	60.2
Sleep disorders	79	36.7
HTA	60	28.2
Sinusitis	57	26.9
Allergic dermatitis	44	20.5
Nutritional status (n = 215)**		
Lean	31	14.4
Normal	94	43.7
Overweight	56	26.1
Obese	34	15.8
**FEV**_**1**_, % (n = 215)***		
Normal	75	34.9
Abnormal	140	65.1
Serum vitamin D (n = 207)****		
Normal	10	4.8
Abnormal	197	95.2
**Sensitization**		
House dust mites (n = 215)	83	38.6
Cat dander (n = 215)	53	24.7
Dog dander (n = 215)	38	17.7
Moulds (n = 182)	15	8.2
Egg yolk (n = 181)	12	6.6
**Simultaneous sensitization to cat dander and dust mites (n = 215)**		
No	176	81.9
Yes	39	18.1

*An ACT score ≥20 indicated "controlled" asthma and a score ≤19 indicated "uncontrolled" asthma.

**Nutritional status was assessed by calculating the body mass index (BMI). A participant with a BMI of <20 kg/m^2^ was considered "lean", 20–24.9 kg/m^2^ "normal", 25–29.9 kg/m^2^ "overweight", and ≥30 kg/m^2^ "obese".

***A respondent with a maximum FEV_1_ >80% was considered to have "normal" lung function and a FEV_1_ ≤80% or less was considered to have "abnormal" lung function.

**** Serum vitamin D levels ≥30 ng/ml were considered "normal" and those <30 ng/ml were considered "abnormal”.

### Univariable associations

In univariate analysis (Table 3), we found that asthmatics aged at least 50 years, with a low level of education, a low socioeconomic status, and who smoke (or had stopped smoking for less than six months) more frequently had uncontrolled asthma. Additionally, those sensitized to both cat dander and *Blomia tropicalis*, with no family atopy, an abnormal FEV_1_, as well as those who suffer from heartburn, sleep disorders, and hypertension also more frequently had uncontrolled asthma. However, the distribution of asthma control was not different by gender, nutritional status, serum vitamin D level, or comorbidities such as allergic rhinitis, sinusitis and allergic dermatitis.

**Table 3:** Associations between potential risk factors and asthma control.

Potential risk factors	Uncontrolled asthma		Controlled asthma		Chi-square test	p
	n	%	n	%		
**Age**					**7.396**	**0.026**
< 25 years	12	37.5	20	62.5		
25 to 49 years	46	53.5	40	46.5		
≥ 50 years	63	64.3	35	35.7		
**Gender**					0.260	0.610
Male	33	58.9	23	41.1		
Female	88	55.0	72	45.0		
**Educational status**					**10.609**	**0.005**
Low	57	63.3	33	36.7		
Medium	44	61.1	28	38.9		
High	20	37.0	34	63.0		
**Socioeconomic status**					**6.833**	**0.033**
Low	57	66.3	29	33.7		
Medium	24	55.8	19	44.2		
High	40	46.5	46	53.5		
**Family history of atopy**					**7.952**	**0.005**
No	64	66.7	32	33.3		
Yes	57	47.5	63	52.5		
**Smoking**					**5.354**	**0.021**
No / Ex-smoker ≥ 6 months	99	52.9	88	47.1		
Yes / Ex-smoker < 6 months	22	75.9	7	24.1		
**Simultaneous sensitization to dust mites and cat dander**					**4.661**	**0.031**
No	93	52.8	83	47.2		
Yes	28	71.8	11	28.2		
**Nutritional status**					1.933	0.380
Obese/overweight	55	61.1	35	38.9		
Normal	48	51.1	46	48.9		
Lean	18	58.1	13	41.9		
**Serum vitamin D level**					2.779	0.095
Normal	3	30.0	7	70.0		
Abnormal	112	56.9	85	43.1		
**FEV**_**1**_ **(%)**					**7.058**	**0.008**
Normal	33	44.0	42	56.0		
Abnormal	88	62.9	52	37.1		
**Heartburn**					**4.038**	**0.044**
No	41	47.7	45	52.3		
Yes	80	61.5	50	38.5		
**Allergic rhinitis**					0.742	0.389
No	36	61.0	23	39.0		
Yes	85	54.5	71	45.5		
**Sleep disorders**					**3.871**	**0.049**
No	69	51.0	67	49.0		
Yes	51	64.6	28	35.4		
**Hypertension**					**4.886**	**0.027**
No	79	51.6	74	48.4		
Yes	41	68.3	19	31.7		
**Sinusitis**					0.206	0.650
No	87	56.1	68	43.9		
Yes	30	52.6	27	47.4		
**Allergic Dermatitis**					0.361	0.548
No	98	57.3	73	42.7		
Yes	23	52.3	21	47.7		

There was a positive correlation between the level of education and the socioeconomic status (r = 0.539, p < 0.001). There was a clear trend from “low to high” for educational and socioeconomic status in this study (See S1 Table).

### Multivariable analysis

In multivariable analysis (Table 4), seven factors were associated with uncontrolled asthma: male, low level of education, sensitization to both cat dander and dust mites, an FEV_1_ ≤80%, heartburn, sleep disorders, and abnormal serum vitamin D levels. Male asthmatics were over twice as likely to have uncontrolled asthma as female ones (aOR 2.24; 95% CIs 1.04–4.79). Asthmatics with a low level of education were over three times more likely to have uncontrolled asthma than those with high level of education (aOR 3.23; 95% CIs 1.54–6.67). Subjects with abnormal FEV_1_ were twice as likely to have uncontrolled asthma as those with normal FEV_1_ (aOR 2.07; 95% CIs 1.08–3.96). Asthmatic patients suffering from heartburn or sleep disorders were also twice as likely to have uncontrolled asthma as those who did not (respectively aOR 2.02; 95% CIs 1.04–3.92 and aOR 1.96; 95% CIs 1.04–3.71). Asthmatics sensitive to both cat dander and dust mites were almost three times more likely to have uncontrolled asthma than those who were not (aOR 2.67; 95% CIs 1.16–6.14), while patients with abnormal serum vitamin D levels were over five times more likely to have uncontrolled asthma than those with normal serum vitamin D levels (aOR 5.11; 95% CIs 1.17–22.33).

**Table 4:** Factors associated with uncontrolled asthma in adults in Kinshasa.

Characteristics	Unadjusted OR (95% CIs)	p	Full model		Final model	
			Adjusted OR* (95% CIs)	p	Adjusted OR** (95% CIs)	p
**Age**						
≥ 25 years	1		1			
25–49 years	1.91 (0.83–4.40)	0.125	2.17 (0.79–5.98)	0.133		
50 years and above	3.00 (1.31–6.86)	**0.009**	1.36 (0.46–4.04)	0.582		
**Gender**						
Female	1		1		1	
Male	1.17 (0.63–2.18)	0.610	1.79 (0.80–4.01)	0.159	2.24 (1.04–4.79)	**0.039**
**Educational status**						
Low	2.94 (1.45–5.88)	**0.003**	1.30 (0.57–2.95)	0.529	3.23 (1.54–6.67)	**0.002**
Medium	0.91 (0.48–1.72)	0.772	0.52 (0.18–1.45)	0.211		
High	1		1			
**Socioeconomic status**						
Low	1		1			
Medium	1.45 (0.70–3.03)	0.320	1.24 (0.51–3.00)	0.640		
High	2.26 (1.22–4.18)	**0.009**	1.84 (0.77–4.35)	0.167		
**Smoking**						
No, or ex-smoker of > 6 months	1		1			
Yes, or ex-smoker of < 6 months	2.80 (1.14–6.86)	**0.025**	1.91 (0.70–5.23)	0.208		
**Serum vitamin D level**						
Normal	1		1		1	
Abnormal	3.07 (0.77–12.24)	0.111	5.08 (1.09–23.73)	0.039	5.11 (1.17–22.33)	**0.030**
**FEV1**						
Normal (> 80%)	1		1		1	
Abnormal (≤ 80%)	2.15 (1.22–3.81)	**0.008**	2.27 (1.12–4.61)	0.024	2.07 (1.08–3.96)	**0.029**
**Heartburn**						
No	1		1		1	
Yes	1.76 (1.01–3.05)	**0.045**	1.83 (0.89–3.78)	0.102	2.02 (1.04–3.92)	**0.037**
**Family history of atopy**						
No	1		1			
Yes	0.45 (0.26–0.79)	**0.005**	0.59 (0.30–1.17)	0.131		
**Sleep disorders**						
No	1		1		1	
Yes	1.77 (0.99–3.13)	0.050	2.33 (1.18–4.58)	0.015	1.96 (1.04–3.71)	**0.039**
**Hypertension**						
No	1		1			
Yes	2.02 (1.08–3.79)	**0.028**	1.23 (0.55–2.77)	0.613		
**Simultaneous sensitization to dust mites and cat dander**						
None	1		1		1	
Cat dander and dust mites	2.27 (1.06–4.84)	**0.034**	2.32 (0.98–5.50)	0.056	2.67 (1.16–6.14)	**0.021**

*: the full model: kept all variables in the model using enter method

**: age, gender, educational status, socioeconomic status, history of smoking, family history of atopy, sensitization to both cat dander and dust mites, serum vitamin D level, sleep disorders, hypertension, heartburn and FEV_1_. Backward selection was the method used for the final model: we fit the full model on all explanatory variables; only the significant explanatory variables were kept in the final model

## Discussion

Here we report that just over half of subjects in this sample of adult asthmatics in Kinshasa had uncontrolled asthma (56%). We found that male, low educational level, sensitization to at least two allergens, decreased FEV_1_, heartburn, sleep disorders, and abnormal serum vitamin D level were significantly associated with uncontrolled asthma.

By applying the ACT, 56% of our reported asthmatics had uncontrolled asthma. Although this prevalence seems high, it is consistent with the high trends reported in several studies. Analysis of epidemiological surveillance data from five European countries (France, Germany, Italy, Spain, and the United Kingdom; 2006 European National Health and Wellness Survey, ENHWS) after ten years of application of the Global Initiative for Asthma guidelines revealed an uncontrolled asthma prevalence of 50.4% [10], which rose to 53.5% in the 2010 ENHWS [14]. A multicentre study of Turkish asthma patients in tertiary care reported a 48.5% uncontrolled asthma prevalence [19]. Other studies have reported very high uncontrolled asthma prevalences: 71.9% in the Jilin province in China [20], 68.1% in Saudi Arabia [14], and 71.4% in Ethiopia [21]. In Cameroon, a central African country closer to the DRC, ACTs performed in adult asthmatics seen in specialized consultation revealed an uncontrolled asthma prevalence of 42%, lower than our results [13]. Poor asthma control exposes people to an increased risk of exacerbations, emergency room visits, hospital admissions, and even death [10].

While many studies have found either no association between sex and uncontrolled asthma [22], or association between female and uncontrolled asthma [13, 14, 19, 23, 24, 25]; we found that male were at greater risk of uncontrolled asthma, may be due to a residual confounding as Peters et al have reported in huge sample in USA [26]. There might exist a variable such as treatment adherence, we did not assess in this study which can induce confounding.

The association between low educational level and uncontrolled asthma in the current study has been previously reported worldwide and even in low income countries [27]. Low educational level exposes to less information about asthma prevention and control and lack of self-management education. In this study sleep disorders are associated to uncontrolled asthma. This observation is in concordance with the work by Kavanagh et al. who have found a significant relationship between asthma and sleep disorders due to circadian variation of airway inflammation, suggesting the relevance of continuous positive airway pressure (CPAP) treatment in asthmatic patients with obstructive sleep apnoea disease [28].

After grouping allergen sensitization, we found that participants sensitized to both dust mites and cat dander, the allergens most frequently implicated in asthma [29], were more at risk for uncontrolled asthma than non-sensitized participants. This observation is consistent with the results of Agodokpessi et al. [30], who also reported an association between polysensitization and a high prevalence of uncontrolled asthma. Similarly, investigators of the Trousseau Asthma Program cohort defined several allergic asthma phenotypes, including asthma with unique sensitization to mites with a favourable prognosis (mild persistent asthma) in contrast to early-onset allergenic multisensitization characterized by a higher risk of lung function decline [31]. Asthma control was also significantly associated with an abnormal FEV_1_ ≤ 80%, consistent with the results of Turktas et al. [19]. Poorly controlled asthma is associated with a greater variability in lung function than well controlled asthma [1], and a low FEV_1_ is a powerful predictor of the risk of exacerbations [1].

In this study, there was a trend to a higher proportion of participants with uncontrolled asthma in the vitamin D-deficient group (56.9% vs. 43.1%). Indeed, Korn et al. [32] showed that patients with uncontrolled asthma had lower serum vitamin D levels compared to subjects with better control, while Columbo et al. [15] observed lower basal serum vitamin D levels in asthmatics over 65 years of age with poor disease control compared to a group with good control. Furthermore, in this study, 12 months of vitamin D supplementation improved control as evaluated by the ACT. However, there is still no good-quality evidence that vitamin D supplementation improves asthma control or reduces asthma exacerbations. Further studies are needed [1, 33]. Vitamin D has many immunomodulatory effects. It has been shown to inhibit the release of IL-12 by dendritic cells, affecting thus the T lymphocyte differentiation and deregulating the Th1/Th2 balance. Hypovitaminosis D then favours the overexpression of Th1 cytokines and inhibits the production of anti-inflammatory cytokines such as IL-10. Therefore, abnormal vitamin D level could have potentially harmful health effects and might partly explain the increased prevalence of chronic and allergic diseases world [34].

There are some limitations to this study. The relatively small sample size probably failed to reveal some associations. The recruitment of patients on the basis of reported asthma may have led to selection bias. In this study, evaluation of confounding was particularly relevant in the identification of factors associated with uncontrolled asthma. Most of the confounders were in fact controlled for during the data analysis stage, using multivariate analyses. Even if we had used a multivariate technique, there can remain residual confounding in this study. There was the probability that the additional confounding factors were not considered, because data on these factors were not collected such as treatment adherence. Another source of the error is the classification of subjects with respect to confounding variables. History of smoking, family atopy, sleep disorders, hypertension and heartburn were measured based on declaration of respondents. Nevertheless, the study has the merit of reporting the prevalence of uncontrolled asthma in adult asthmatics in a new population in a low-income country and describes possible correlations between the epidemiological, clinical, functional, and biological aspects of asthma and its control. These preliminary observations will allow the development of more extensive country-specific protocols and policy.

## Conclusions

Here we report that a significant proportion of adult asthmatics in Kinshasa have uncontrolled asthma. Male, low educational level, simultaneous sensitization to cat dander and house dust mites, decreased FEV_1_, heartburn, sleep disorders, and abnormal serum vitamin D level are significantly associated with uncontrolled asthma. These observations are important to consider when developing context-specific intervention strategies.

## Supporting information

S1 Table This is the correlation between level of education and socioeconomic status. (DOCX)

S1 Dataset This is the data used for this article. (DTA)
