# Supplementary material for: Factors associated with uncontrolled asthma in adult asthmatics in Kinshasa, Democratic Republic of Congo
Source: PLoS One. 2019 Apr 18;14(4):e0215530. doi: 10.1371/journal.pone.0215530 (PMC6472784; doi:10.1371/journal.pone.0215530)
Supplement: S1 Table — (DOCX) [file pone.0215530.s001.docx]

**S1 Table. Correlation between level of education and socioeconomic status**

| Education | Low  (n = 86) | | Medium  (n = 43) | | High  (n = 86) | |
| --- | --- | --- | --- | --- | --- | --- |
|  | N | % | n | % | n | % |
| Low (n = 90) | 59 | 65.6 | 17 | 18.9 | 14 | 15.5 |
| Medium (n = 71) | 24 | 33.8 | 16 | 22.5 | 31 | 43.7 |
| High (n = 54) | 3 | 5.6 | 10 | 18.5 | 41 | 75.9 |
